# Sensing Apps and Public Data Sets for Digital Phenotyping of Mental Health: Systematic Review

**DOI:** 10.2196/28735

**Published:** 2022-02-17

**Authors:** Jean P M Mendes, Ivan R Moura, Pepijn Van de Ven, Davi Viana, Francisco J S Silva, Luciano R Coutinho, Silmar Teixeira, Joel J P C Rodrigues, Ariel Soares Teles

**Affiliations:** 1 Laboratory of Intelligent Distributed Systems Federal University of Maranhão São Luís Brazil; 2 Health Research Institute University of Limerick Limerick Ireland; 3 NeuroInovation & Technological Laboratory Federal University of Delta do Parnaíba Parnaíba Brazil; 4 College of Computer Science and Technology China University of Petroleum (East China) Qingdao China; 5 Instituto de Telecomunicações Covilhã Portugal; 6 Federal Institute of Maranhão Araioses Brazil

**Keywords:** mental health, digital phenotyping, sensing apps, data sets, sensor data

## Abstract

**Background:**

Mental disorders are normally diagnosed exclusively on the basis of symptoms, which are identified from patients’ interviews and self-reported experiences. To make mental health diagnoses and monitoring more objective, different solutions have been proposed such as digital phenotyping of mental health (DPMH), which can expand the ability to identify and monitor health conditions based on the interactions of people with digital technologies.

**Objective:**

This article aims to identify and characterize the sensing applications and public data sets for DPMH from a technical perspective.

**Methods:**

We performed a systematic review of scientific literature and data sets. We searched 8 digital libraries and 20 data set repositories to find results that met the selection criteria. We conducted a data extraction process from the selected articles and data sets. For this purpose, a form was designed to extract relevant information, thus enabling us to answer the research questions and identify open issues and research trends.

**Results:**

A total of 31 sensing apps and 8 data sets were identified and reviewed. Sensing apps explore different context data sources (eg, positioning, inertial, ambient) to support DPMH studies. These apps are designed to analyze and process collected data to classify (n=11) and predict (n=6) mental states/disorders, and also to investigate existing correlations between context data and mental states/disorders (n=6). Moreover, general-purpose sensing apps are developed to focus only on contextual data collection (n=9). The reviewed data sets contain context data that model different aspects of human behavior, such as sociability, mood, physical activity, sleep, with some also being multimodal.

**Conclusions:**

This systematic review provides in-depth analysis regarding solutions for DPMH. Results show growth in proposals for DPMH sensing apps in recent years, as opposed to a scarcity of public data sets. The review shows that there are features that can be measured on smart devices that can act as proxies for mental status and well-being; however, it should be noted that the combined evidence for high-quality features for mental states remains limited. DPMH presents a great perspective for future research, mainly to reach the needed maturity for applications in clinical settings.

## Introduction

### Background

Mental health issues have a high prevalence, with 1 in 10 people worldwide experiencing them at any one time [1] and common mental disorders such as depression being closely linked to suicide [2]. Mental disorders are “generally characterized by some combination of abnormal thoughts, emotions, behavior and relationships with others” [3]. Examples are depression, schizophrenia, excessive anxiety and stress, disorders caused by drug and alcohol abuse, and personality and delusional disorders. These disorders pose a significant burden on societies, both emotionally and financially. For example, the cost of mental health disorders in the European Union is estimated at €600 billion (~US $451 billion), or 4% of gross domestic product [4]. COVID-19 has had a further negative impact on global mental health [5].

Mental disorders are usually diagnosed exclusively on the basis of symptoms, which are identified from patients’ interviews and self-reported experiences. Sometimes these experiences are gathered using ecological momentary assessment (EMA) solutions [6], but mostly therapists rely on patients remembering such experiences during sessions. EMA solutions are used as a research method to collect, at fixed or random moments, reports from individuals about perceptions of their behaviors and feelings, and what they have done or experienced. It is well known that the intervening time and current state of the patient bias his/her memory of the experience. In addition, biological tests to assist diagnosis remain hard to be developed [7]. Based on the need to develop solutions able to objectively diagnose and monitor mental health, different solutions have been proposed, such as mobile apps [8,9] and machine learning (ML) solutions [10], which are even more indicated today due to the global pandemic situation [11,12]. Digital phenotype solutions are examples that can expand the ability to identify and diagnose health conditions from the interactions of people with digital technologies [13]. Specifically, digital phenotyping of mental health (DPMH) [14] seems to be a promising approach not only to deal with the problem of diagnosing the issue, but also to be applied to the treatment.

The omnipresent adoption of pervasive devices, including smartphones and wearable sensors, provides novel opportunities for tracking mental health status and disorders. Digital phenotyping refers to the “moment-by-moment quantification of the individual-level human phenotype in-situ using data from smartphones and other personal digital devices” [15], thereby removing limitations created by the aforementioned bias in self-reports.

DPMH solutions require collecting and analyzing large amounts of different types of social and behavioral data that can represent experiences of the users and their interactions with people, places, and devices. These context data can be passively gathered, for instance, from ubiquitous sensors, social media, and health care systems [16]. After collection, pieces of raw data are usually preprocessed and transformed into useful data or data sets to be mined [17]. For example, these data sets may be analyzed or used as input to build ML models [18], including for DPMH, to produce valuable insights and evidence. Therefore, DPMH sensing apps are primarily responsible for collecting and preprocessing data, with the data sets produced being important for developing such models. This study systematically reviews the sensing apps and data sets for DPMH.

### Definitions

In the last few years, the number of smart devices, that is, mobile (eg, smartphone, tablet) and wearable (eg, smart band, smartwatch) devices, has grown globally. They have enabled the development of research in the health area, including mental health [10]. The term “digital phenotype”, defined by Jain and colleagues [13], refers to the identification of human behavior patterns, whereas “digital phenotyping” is a monitoring approach that can collect patients’ behavioral markers passively [19]. Therefore, DPMH solutions aim at collecting multimodal pieces of information from digital devices using sensing apps to combine them with electronic medical records to objectively contribute to the identification of symptoms of mental disorders. In this context, sensing apps are tools for mobile and wearable devices used to collect useful user information.

**Figure 1:**
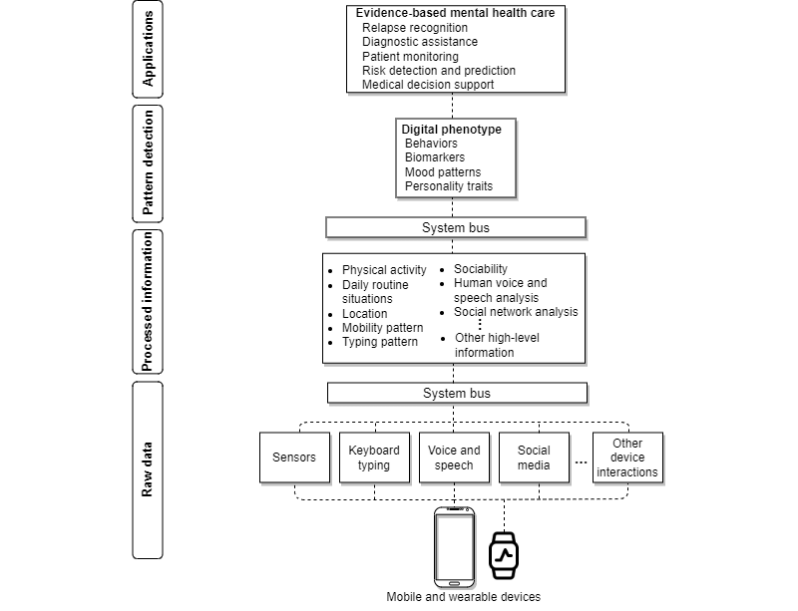
The process of digital phenotyping.

Our vision of the digital phenotyping process organized in layers is presented in Figure 1. The process starts at the first layer with the collection of raw data from different sources (eg, global positioning system [GPS] sensors, keyboard inputs, voice, and social media). These data can be collected both actively, in which user inputs are explicitly required, and passively [20], which only requires the user’s permission to access context data. In the next layer, these data are processed to provide high-level information. High-level information represents not only human behaviors (eg, sociability, physical activity) and habits (eg, mobility, sleep) but also other information of interest for professionals (eg, environmental context, mood). Next, human behavioral patterns that compose digital phenotypes (eg, biomarkers, mood patterns) can be recognized using computational tools (eg, ML, data mining, statistical models). Finally, we visualize the application layer, which corresponds to digital phenotypes used by health professionals for evidence-based mental health care.

### Related Work

Since the aforementioned concepts were proposed in the literature, many research studies have been performed. For this reason, researchers have also reviewed different aspects regarding this research topic. Table 1 presents a list composed of related reviews.

**Table 1:** List of related review articles.

Study	Description
Garcia-Ceja et al [10]	A survey on mental health monitoring using mobile and wearable sensors focused on multimodal sensing and machine learning solutions.
Cornet and Holden [21]	An SLR^a^ on passive sensing using specifically smartphones focused on health and well-being.
De-La-Hoz-Franco et al [22]	An SLR aimed at finding data sets composed of sensor data for human activity recognition.
Trifan et al [23]	This SLR aimed to identify studies on the passive use of smartphones for generating outcomes related to health and well-being. It identified that one of the areas most explored by mobile passive sensing is mental health.
Seppälä et al [24]	An SLR on mobile solutions focused on uncovering associations between sensor data and symptoms of mental disorders (ie, behavioral markers).
Liang et al [14]	A comprehensive survey addressing different topics on DPMH^b^.
Benoit et al [20]	This SLR sought to map DPMH tools that use machine learning algorithms across the schizophrenia spectrum and bipolar disorders.
Antosik-Wójcińska et al [25]	This work presents an overview of studies about smartphone systems focused on monitoring or detecting bipolar disorder.

^a^SLR: systematic literature review.

^b^DPMH: digital phenotyping of mental health.

This review differs from the previous ones in the following aspects: First, instead of focusing on a specific mental state/disorder, this review presents an overview of how different types of devices and detection modalities have been used to monitor a wide variety of different mental states within the DPMH area. Second, this review covers not only active collection solutions, which are emphasized in most reviews, but also passive sensing proposals. Third, this review focuses on the technical features of sensing apps and data sets (eg, size, sensors used to collect data, and types of context data). Technical features can be identified to serve as a basis for the use or development of new apps (eg, physical and virtual sensors used to collect data, operating systems for which the apps were developed, types of context data collected, inferred information). Finally, not all previous reviews were conducted systematically. Our article therefore provides researchers with an overview of the available technological framework for DPMH and can serve as a preliminary guide for current and further research.

### Objectives and Research Questions

This systematic review intends to provide a technical characterization and summary of sensing apps and public data sets for DPMH. By “public” we mean data sets that are available for free download for use in other research endeavors. These 2 topics (ie, sensing apps and public data sets) are jointly addressed in this review as complementary content. When researchers do not have access to DPMH data sets, they need sensing apps. This paper therefore can be a starting point not only to gain knowledge on the current sensing apps for DPMH (which consequently enables the development of new solutions), but also to find reusable ones. Therefore, the objectives of this article are to (1) present results from a systematic search on digital libraries and data set repositories, and then identify and categorize them by considering their characteristics; (2) summarize their main features (measurable pieces of data that can be used for analysis or creation of ML models, such as data collection time stamp, context data produced by DPMH solutions, and data self-reported by users), which are useful for researchers, either mental health or information technology ones, to conduct further investigation and comment on their usefulness; and (3) identify trends in and research opportunities for DPMH. Results of this systematic review are also relevant for data engineers and ML specialists who make efforts in developing DPMH solutions.

To achieve the objectives of this systematic review, we defined the following research questions for sensing apps (SA-RQs) and data sets (DS-RQs):

SA-RQ1: What context data are collected through DPMH sensing apps?

SA-RQ2: What high-level information can be inferred from the context data collected by DPMH sensing apps?

SA-RQ3: How is the identified high-level information used to support mental health?

DS-RQ1: What features are available in public data sets for DPMH?

DS-RQ2: What high-level information can be derived from public data sets for DPMH?

## Methods

### Design

This study was conducted based on the guidelines for systematic literature reviews in software engineering proposed by Kitchenham and Charters [26]. This review followed 3 main phases: planning, conducting research, and dissemination of results. These phases were supported by the *Parsif.al* [27] tool, which provides an online shared work environment for planning and executing systematic reviews. In this section, we present how this review was planned and conducted.

### Search Strategy

The search aimed to identify data sets and studies that have presented sensing apps capable of collecting data. Two (JM and IM) researchers conducted an exhaustive search on January 14, 2021, on data set repositories and digital libraries. The search for data sets was performed in 20 repositories (Multimedia Appendix 1). The search for articles reporting sensing apps was conducted in the following digital libraries: ACM Digital Library, DOAJ, IEEE Xplore, Web of Science, PubMed, PsycInfo, ScienceDirect, and Scopus. These databases were selected because they collect reliable studies related to mental health informatics.

We designed the search strings to retrieve data sets and articles presenting sensing apps for DPMH (Table 2). These search strings were carefully designed to meet the research focus. In the string to search data sets, we defined the 2 main terms (ie, mental health and digital phenotyping) and decided to use Boolean “OR” as the link for them to get comprehensive results. The search string for articles was developed based on the review objective, research questions, and their motivations. We used keywords and their synonyms to maximize results. To avoid missing papers, we evaluated the suitability of the string in a pilot search, in which we used those studies developed by Liang et al [14] (ScienceDirect) and Torous et al [15] (PubMed) as control articles. This pilot search was able to retrieve the cited studies, thus demonstrating its ability to find articles relevant for this review. At the end of the search, duplicate data sets and articles were identified and removed using the *Parsif.al* tool.

**Table 2:** Keywords and their synonyms.

Search	Source	String
Data sets	Data set repositories	“mental health” OR “digital phenotyping”
Sensing apps	Digital libraries	(“mental health” OR “mental disorder*” OR “mental illness” OR “mental state” OR “mental disease”) AND (“mobile device” OR “smartphone*” OR “wearable device*” OR “sensor*” OR “wearable*” OR “mobile application*” OR “mobile health” OR “mHealth” OR “mobile phone*” OR “sensor data”) AND (“passive detection” OR “data collection” OR “digital phenotype” OR “digital phenotyping” OR “digital health” OR “monitoring” OR “passive sensing”)

### Selection Criteria

A set of selection criteria was defined to track research articles and data sets. Textbox 1 presents the selection criteria for scientific studies with sensing apps and data sets. Importantly, no date range limits were applied to the literature included in the review. In the selection of scientific articles, criterion EC1 excluded studies presenting the development of EMA apps, and papers that do not present a new DPMH solution (eg, studies using a DPMH solution previously described/published in another paper). For data set selection, criterion EC1 excluded those data sets that were not publicly available, that is, those protected and not accessible to be reused by other researchers.

In the selection phase, 2 researchers (JM and IM) performed the data set selection process based on the inclusion and exclusion criteria. In a second step, the same 2 researchers independently performed the study selection process. This process consisted of 3 sequential phases: (1) study screening by means of metadata analysis (ie, title, abstract, and keywords); (2) full-text analysis of the articles selected in the screening phase; and (3) conducting backward snowballing [28]. Next, the level of agreement between the selections was calculated using the Cohen κ coefficient [29]. In the end, the 2 researchers conducted discussions to resolve selection conflicts and, when there was no consensus, judges (2 other authors, namely, AT and DV) deliberated on the disagreements.

**Selection criteria.**

**Inclusion criteria (IC)**

*Scientific articles*

IC1: Primary studies that present pervasive solutions to collect data for digital phenotyping of mental health.

IC2: Full papers.

IC3: Papers in English language.

*Data sets*

IC1: Available to be downloaded and used in other research studies (ie, public data set).

IC2: Focused on mental health or specific mental disorders.

IC3: Relevant data (eg, behavioral, physiological, social) for mental health collected through pervasive technologies.

IC4: Content in English language.

**Exclusion criteria (EC)**

*Scientific articles*

EC1: Articles presenting research on digital phenotyping of mental health without involving a proposal of a pervasive solution.

EC2: Gray literature.

EC3: Articles that have other publications with a more current and complete version of the proposed solution.

*Data sets*

EC1: Not publicly available.

EC2: With no content related to mental health.

EC3: Data on treatments of patients with mental disorders without using pervasive devices.

EC4: Content in languages different from English.

EC5: Online surveys on ethnographic characteristics and prevalence of mental disorders.

EC6: Composed exclusively by multimedia data (eg, video, audio) or electroencephalography data.

### Data Extraction

In this step, data were extracted from the selected articles and data sets to answer the research questions defined in this review. For this purpose, a data extraction form was designed by 2 authors (JM and IM) and validated by the judges. Specifically, we designed the items in the form to extract relevant information presented by the reviewed studies and data sets, thus enabling us to answer the research questions, and identify open issues and research trends. Multimedia Appendix 2 presents the items in the data extraction form.

## Results

### Study Selection

**Figure 2:**
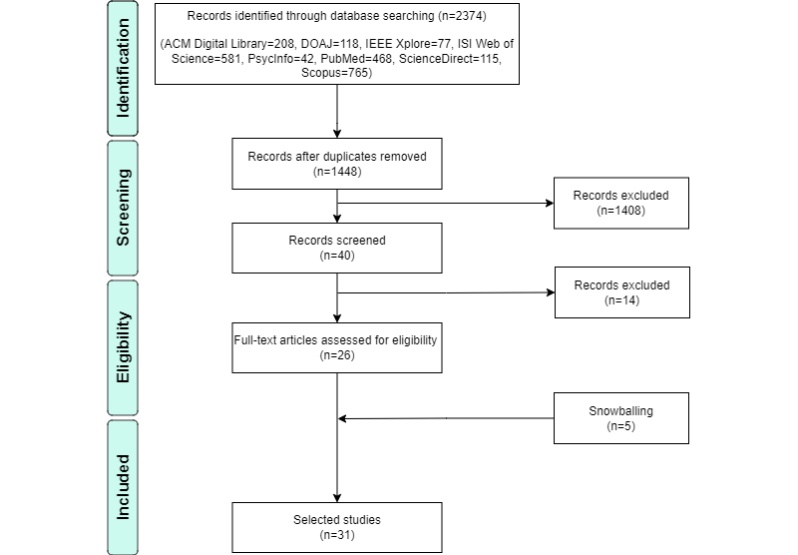
PRISMA-based flowchart describing the selection of studies.

An overview of the review process with results is presented in Figures 1 and 2. In Figure 2, 8 digital libraries were used to search for scientific articles that presented sensing apps for DPMH. A total of 2374 articles were returned. We removed 926 duplicate articles. The inclusion and exclusion criteria from Textbox 1 were applied to select 26 selected studies. The Cohen κ statistical test showed an agreement level of ≈0.87 between researchers, which is considered an almost perfect agreement [29]. Next, researchers used the 1-level backward snowballing approach and added 5 articles. This resulted in 31 articles for inclusion in the data extraction process.

**Figure 3:**
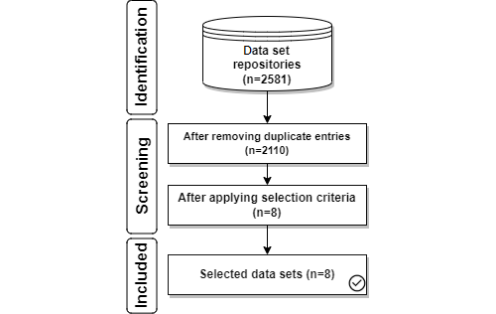
Flowchart describing the selection of data sets.

In Figure 3, 20 data set repositories were searched to return 2581 data sets with 471 duplicates that were removed. After applying selection criteria (Textbox 1) and resolving conflicts, 8 data sets remained for analysis.

### Sensing Apps

Table 3 summarizes the 31 apps identified, which are presented in ascending order by year of publication. Multimedia Appendix 3 presents the full version of the table. Context data sources are categorized as follows to present the sensors used by the apps based on the work by Palaghias et al [30]: ambient (eg, microphone, camera), positioning (eg, GPS, Wi-Fi), virtual (eg, phone calls, SMS text messages), and inertial (eg, accelerometer, gyroscope). Table 3 also presents high-level information inferred and types of analyses performed on the collected data. Apps that do not infer information (ie, defined as “It does not infer information”) are only intended to collect data from smart devices. In this case, collected data are usually sent to servers for analysis. These apps are flagged as “Raw data collection” in Table 3.

**Table 3:** Summary of reviewed sensing apps.

App	Context data source	High-level information	Type of analysis
Funf [31]	Positioning, inertial, and virtual	It does not infer information	Raw data collection
Mobilyze [32]	Positioning, inertial, virtual, and ambient	Mood, emotions, cognitive/motivational states, physical activity, social context	Mental state prediction
Purple Robot [33]	Positioning, inertial, and virtual	It does not infer information	Raw data collection
AWARE [34]	Positioning, inertial, and virtual	It does not infer information	Raw data collection
Sensus [35]	Positioning, inertial, virtual, and ambient	It does not infer information	Raw data collection
MOSS [36]	Positioning and virtual	Physical activity, mobility, device usage, sociability, app usage	Mental state classification
Beiwe [15]	Positioning, inertial, virtual, and ambient	It does not infer information	Raw data collection
EVO [37]	Positioning, inertial, and virtual	It does not infer information	Raw data collection
CrossCheck [38]	Positioning, inertial, virtual, and ambient	Sleep, sociability, mobility, physical activity, device usage	Mental state prediction
SituMan [39]	Positioning and inertial	Daily routine situations (eg, working, studying)	It recognizes daily routine situations using fuzzy logic
EmotionSense [40]	Positioning, inertial, virtual, and ambient	Semantic locations, physical activity, sociability	Correlation analysis and mental state classification
StudentLife [41]	Positioning, inertial, virtual, and ambient	Sociability, mobility, physical activity, device usage	Correlation analysis
Undefined [42]	Positioning, inertial, and ambient	Physical activity, mobility, and sociability	Correlation analysis
AMoSS [43]	Positioning	Mobility	Mental state prediction
eB2 [44]	Positioning and virtual	Mobility	Mental state classification
EARS [45]	Positioning, inertial, virtual, and ambient	It does not infer information	Raw data collection
SleepGuard [46]	Inertial and ambient	Posture/position of body when sleeping	Mental state classification
Moment [47]	Virtual	It does not infer information	Mental state classification
TypeOfMood [48]	Virtual	It does not infer information	Mental state classification
RADAR-base [49]	Positioning, inertial, virtual, and ambient	It does not infer information	Raw data collection
SHADO [50]	Positioning, inertial, and ambient	Physical activity, mobility, sleep, sociability	Correlation analysis and mental state classification
InSTIL [51]	Positioning, inertial, virtual, and ambient	It does not infer information	Raw data collection
Lamp [52]	Positioning	Physical activity	Correlation analysis
SOLVD [53]	Positioning, inertial, virtual and ambient	Mobility, sociability, context of daily life (eg, duration of sleep)	Correlation analysis
STDD [54]	Inertial, virtual, and ambient	Physical activity, mood, sociability, sleep	Mental state classification
Moodable [55]	Positioning, virtual, and ambient	Sociability and mobility	Mental state classification
Cogito Companion [56]	Positioning and Virtual	Mood, stress level, and well-being	Mental state classification
Strength Within Me [57]	Virtual	Sleep, mobility, and sociability	Mental state prediction
EuStress [58]	Ambient	It does not infer information	Mental state prediction
Mood Triggers [59]	Positioning, inertial, virtual, and ambient	Mobility and sociability	Mental state prediction
Data Collector [60]	Positioning and inertial	Physical activity and mobility	Mental state classification

### Data Set Characterization

Table 4 shows the 8 selected data sets in descending order by number of participants. Two of them have sleep quality data: data sets DS1 and DS7, in which the data are derived from activity trackers such as Fitbit, smartwatches, and smartphones. We identified 2 data sets (DS3 and DS5) with data collected from various sensors, which we refer to as multimodal. We identified 2 data sets (DS3 and DS5) that were generated by the StudentLife [41] and Beiwe [15] sensing apps, respectively, shown in Table 3.

**Table 4:** Summary of DPMH data sets.

Data set	Study	High-level information	Features	Device type/operating System	Number of participants	Study duration	Size
DS1^a^ [61]	[62]	Sleep quality	Fitbit data (eg, heart rate, sleep duration, sleep time, wake time)	Watch Fitbit	482	3-11 nights	392.32 KB
DS2 [63]	[64]	Activity	Actigraph (time stamp, activity measurement from the actigraph watch)	Actigraph watch	55	Average 12.6 days	4.3 MB
DS3 [65,66]	[41,67]	Multimodal (stress, sleep, mood, physical activity, sociability, well-being)	Self-report questionnaires, activity, audio, Bluetooth encounters, conversation, lightness, GPS^b^ coordinates, phone charge, screen on/off, Wi-Fi IDs	Smartphone (Android)	48	66 days	230 MB/5 GB
DS4 [68]	[69,70]	Sociability	Self-reports, battery level, Bluetooth encounters	Smartphone (Android, iOS)	32	4 weeks	9.7 MB
DS5 [71]	[15]	Multimodal (mobility, sociability, sleep)	Self-report questionnaires, accelerometer, app logs, Bluetooth encounters, call logs, GPS coordinates, power state, Wi-Fi	Smartphone (Android, iOS)	6	3 months	776.7 MB
DS6 [72]	[73]	Mood, depression symptoms	Self-report questionnaires	Smartphone (Android, iOS)	3	14 days	2.7 MB
DS7 [74]	—	Sleep quality	Start, end, sleep quality, time in bed, wake-up time, sleep notes, heart rate, number of steps	Wearable device and smartphone (iOS)	1	4 years	66.11 KB
DS8 [75]	—	Mood	Self-reported mood	Mobile social network (Twitter app)	1	2 years	131 KB

^a^DS: data set.

^b^GPS: global positioning system.

Data set DS1 [61] presents sleep data (eg, total sleep time and sleep efficiency) obtained from Fitbit Charge HR activity trackers used by 482 individuals [62], while data set DS2 [63] includes actigraphic data collected from patients with unipolar and bipolar disorders and 32 healthy controls [64]. Data set DS3 [65,66] contains data gathered from different sensors and EMA questionnaires collected from smartphones of 48 undergraduate and graduate students over 66 days [41,67]. Data set DS4 [68] comprises Bluetooth device scan, battery level, and EMA data collected at regular intervals for 4 weeks [69,70], while data set DS5 [71] presents passive data (eg, GPS, Wi-Fi, Bluetooth, and accelerometer) and active data (EMA survey responses) collected over 3 months [15]. Data set DS6 [72] contains EMA assessments of depression symptoms using the 9-item Patient Health Questionnaire (PHQ-9) [73]. Data set DS7 [74] presents sleep data collected through the Sleep Cycle mobile app [76]. Finally, data set DS8 [75] presents values extracted from Twitter posts collected from a person using Exist [77] over 2 years.

### Context Data Collected by DPMH Sensing Apps (SA-RQ1)

Sensing apps identified in this review collect context data from mobile and wearable devices to support DPMH. At a high level, the sensors that measure context data can be seen as physical and virtual sensors [78], which generate a diversified set of behavioral data. Physical sensors are hardware components embedded or connected to devices responsible for collecting context data. Some examples are accelerometers to measure user activity, light sensors to measure ambient light levels, and GPS to collect user’s locations. Virtual sensors represent software components capable of recording interactions of individuals with devices or using a number of physical sensors (or other virtual ones) to construct a higher-level feature. Examples of such sensors are social interaction sensors that may use Bluetooth encounters (ie, co-location information between individuals or places), Wi-Fi network, and sound data to infer social activity; and user–device interaction sensor, which measures user interactions with devices (eg, call logs, SMS text messages, app usage, screen on/off).

**Figure 4:**
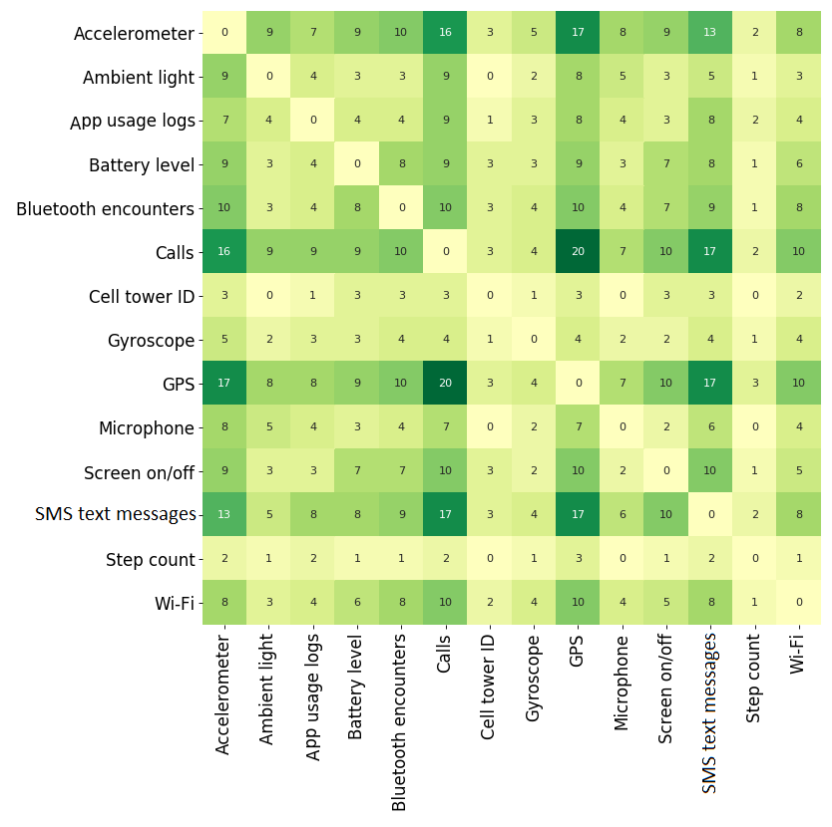
Context data sources used in the reviewed studies. GPS: global positioning system.

Figure 4 presents a heat map of the combination of context data sources for the 31 sensing apps, showing the most used sensors in DPMH solutions. In this analysis, we investigated the frequency of the combination of each type of context data source, highlighting the main sets of sensors explored by the sensing apps. For example, Bluetooth encounters are often combined with accelerometer (n=10), battery level (n=8), calls (n=10), GPS (n=10), screen on/off (n=7), SMS text messages (n=9), and Wi-Fi (n=8), while app usage logs are often combined with accelerometer (n=7), calls (n=9), GPS (n=8), and SMS text messages (n=8). We also identified from this analysis that step count (Fitbit), cell tower ID, and gyroscope are combined less often with other context data sources. The analysis of the combination of context data sources (Figure 4) demonstrates an interest in performing data fusion to identify multiple high-level information and emphasizes the combination of context data sources resulting from the interest in monitoring such information. For example, we identify an interest in recognizing sociability information by combining call logs with Bluetooth encounters (n=10) and SMS text messages (n=17). We also recognize that GPS is often combined with Wi-Fi (n=10) to recognize mobility aspects. In addition, the interest in monitoring multiple high-level information in the same app resulted in different combinations of context data sources. For example, the combination of GPS with call logs (n=20), accelerometer (n=17), and screen on/off (n=10) is a result of an interest in monitoring sociability, physical activity, and device usage patterns, respectively.

### High-Level Information Identified by Sensing Apps (SA-RQ2)

From the context data collected by sensing apps, researchers can extract high-level information representing different types of situations (eg, sociability, mobility). Table 3 presents the situations of interest identified from context data. Sensing apps aimed to identify information related to the physical and environmental aspects of the monitored individuals, such as mobility patterns [38] (eg, places visited, total distance traveled, time spent in locations), physical activities (activity type and duration), daily routine situations (eg, working, studying), and environmental context (eg, ambient temperature).

**Figure 5:**
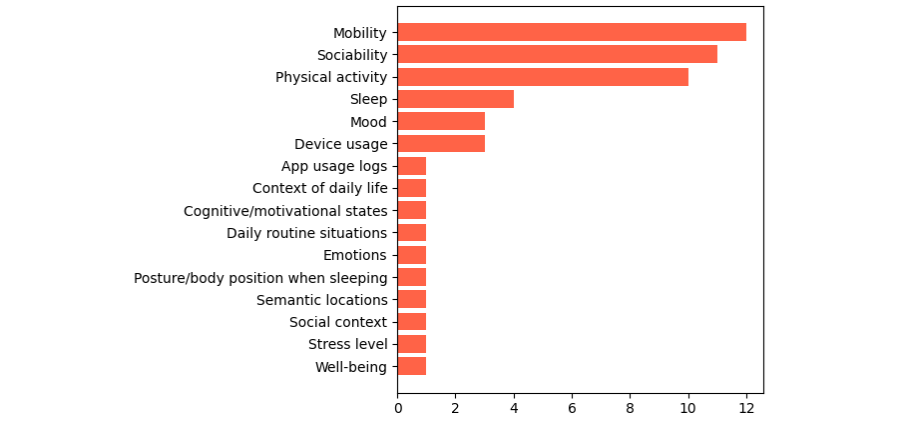
High-level information summary.

Figure 5 shows the types of high-level information generated by the sensing apps. The 3 types of information that stand out are human behavioral patterns related to mobility, sociability, and physical activity (n≥10). Information about the individual’s condition was also derived, such as mood and sleep quality.

Researchers also explored information about device usage, which was derived from logs such as calls, SMS text messages, screen on/off events, and app usage. In general, studies have been able to build apps that achieve promising results of performance metrics (eg, accuracy, sensitivity, specificity) in identifying useful high-level information for mental health professionals. By contrast, there are some researchers developing apps that have not transformed context data into high-level information (ie, they focus only on raw data collection), and these are not shown in Figure 5.

### Support for Monitoring Mental Health (SA-RQ3)

The sensing apps identified used high-level information to provide a variety of mental health services. Table 3 shows the types of analyses performed based on the high-level information identified. Some apps infer daily routine situations and send recommendations in real time [58], thus aiming to provide tools to improve services of health professionals. Most approaches to support mental health monitoring were as follows: correlation, classification, and prediction. Correlation analyses associate features extracted from high-level information with mental states of the monitored individual, that is, they aim to find evidence that identified behaviors have significant correlations with psychological well-being [79]. Researchers also used identified behaviors to design ML models capable of classifying and predicting mental states [32,80], which can be used as decision support tools for health professionals. Lastly, some studies [31,81,82] did not report on additional analyses, but concentrated on describing the features of their sensing apps to facilitate DPMH research.

**Figure 6:**
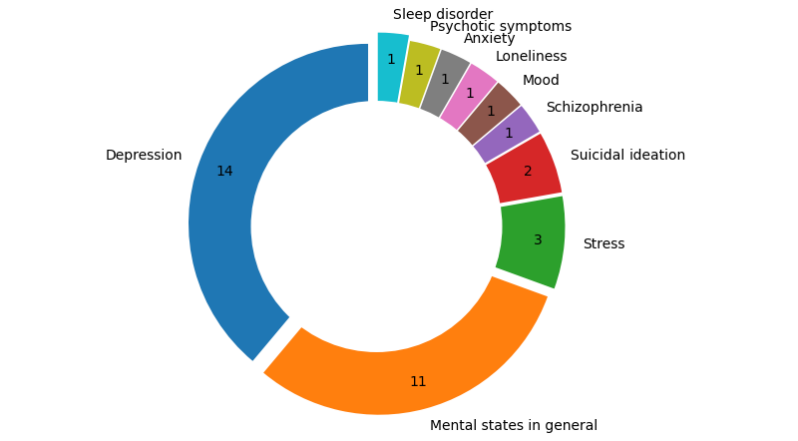
Mental states/disorders targeted by sensing apps.

Figure 6 shows the mental states/disorders studied by DPMH research. Apps classified as “Mental states in general” did not focus on a specific mental disorder; instead, they are generic to be used in studies for different mental health disorders. We found 14 articles with a focus on individuals with depression. Other mental states/disorders are schizophrenia, mood, suicidal ideation, stress, loneliness, anxiety, and psychotic symptoms, all with between 1 and 3 studies returned in our search. We identified 11 articles that did not specifically address a particular mental disorder in their studies.

### Features Available in Data Sets (DS-RQ1)

The selected data sets have several types of features extracted from context data collected by sensing apps. These features model various aspects of human behavior that can be applied to the development process of new tools for monitoring and intervention in mental health. Table 4 presents the features available in the selected data sets. Data sets DS1 and DS7 contain features related to sleep. They provide information such as sleep start and end, sleep quality, time in bed, wake-up, sleep notes. Data sets DS4 and DS8 have features related to the social aspect such as self-reports of social interactions and Bluetooth encounter data, while data sets DS6 and DS8 provide actigraph data and self-reports, respectively. Data sets DS4 and DS5 have features capable of modeling more than 1 human behavior (ie, multimodal), thus providing data from different sources. These sources provide multimodal context data that can be fused to generate meaningful high-level information [10]. Moreover, multimodal data sets can support DPMH research under different aspects of interest for professionals, such as patient’s mobility and sociability.

### Possible High-Level Information Derived From Data Sets (DS-RQ2)

The selected data sets have features capable of modeling different types of human behavior. Therefore, to understand the potential for applying these data to DPMH, we identified high-level information that can be derived from these data sets based on the available context data. Table 4 presents high-level information inferred. Explicitly, these data sets can model the situations listed in Textbox 2.

Additionally, some data sets contain high-level information such as mood, mental status, and mental disorder symptoms. These types of information are self-reported by participants using questionnaires (eg, PHQ-9) and EMA solutions through smart devices.

**Situations modeled by data sets.**

*Sociability*

This can be quantified using context data that allow characterizing social relationships of the participants such as interactions on online social networks, and face-to-face and device-mediated interactions [83]. These data sets contain context data such as posts on social networks, Bluetooth encounters, global positioning system (GPS) coordinates, or conversational activity inferred from microphone signals.

*Physical activity*

This is routinely measured using accelerometer and GPS data, resulting in either a log of user physical activities or an aggregate measure of energy expenditure.

*Sleep*

This is mostly measured in terms of sleep quality and sleep duration of the participants. In general, these data sets have features such as sleep quality, total sleep time, time in bed, and wake-up inferred from contextual data such as heart rate and screen on/off logs, and ambient light.

*Multimodal*

These data sets comprise several types of context data (eg, accelerometer, ambient light, battery level, Bluetooth, GPS, screen on/off, questionnaires [9-item Patient Health Questionnaire]), which allow characterizing more than 1 behavior of the participants such as sociability, mobility, and physical activity.

## Discussion

### Principal Findings

**Figure 7:**
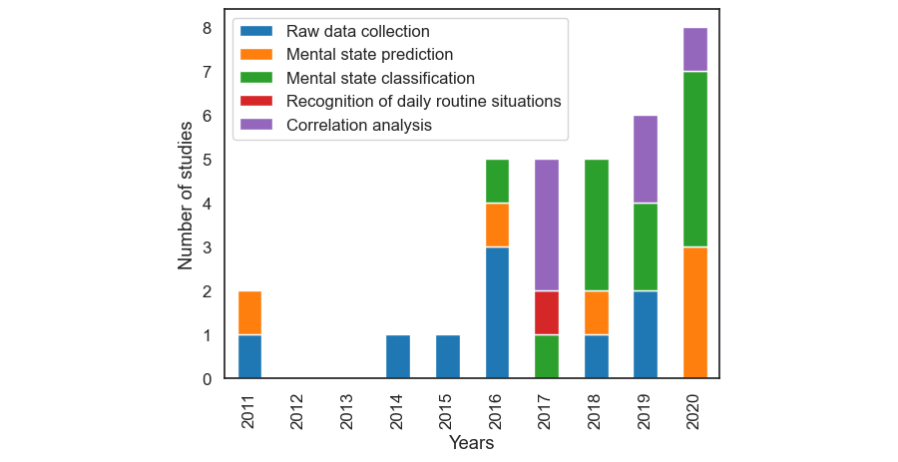
Number of published studies by year and types of analysis.

Our review shows that there are features that can be measured on smart devices that can act as proxies for mental status and well-being, but it should be noted that the combined evidence for high-quality features for mental states remains limited. Researchers have conducted several types of analysis on the data collected. In principle, we recognize a trend to design features from the data collected (Figure 7) to train ML models capable of classifying mental states/disorders (n=11) and predicting future mental states/disorders (n=6). We also note a substantial effort in analyzing correlations between features designed from the collected data and mental states/disorders (n=6). This type of analysis aims to find evidence of the viability and usefulness of DPMH for clinical practice. Furthermore, there are apps that only collect raw context data (n=9) to be analyzed subsequently, and 1 app (SituMan [39]) focused on the recognition of daily routine situations.

The literature mostly reports on the measurement of mobility, sociability, sleep, physical activity, and mood. Mobility represents high-level information derived from the movement sequence of individuals. These patterns are identified by processing GPS and Wi-Fi samples, which allow for the recognition of mobility traces. Sociability is measured using context data sources such as call logs, SMS text messages, Bluetooth encounters, and microphone data. These pieces of data allow identifying physical and virtual social interactions. Sleep information is measured by contextual data fusion such as ambient light, movement activity, screen on/off, and ambient sound. In addition, researchers have used Fitbit data to recognize sleep quality. Physical activity is recognized using data from inertial sensors (eg, accelerometer, magnetometer, gyroscope), making it possible to classify different types of activities such as walking, running, and stationary. Finally, mood has been recognized using different context data sources, such as accelerometer and heart rate monitor of wearable devices, combined with self-reports.

The different ways in which these features are inferred and reported make it impossible to compare results across studies, or combine data sets to achieve greater statistical power. For this reason, we believe the research community would benefit from a clear standard on the measurement of these behaviors. The data sets identified and studies in this review provide an interesting starting point for such consensus building. Particularly, the StudentLife data set [41] has been explored by many studies that propose solutions capable of supporting mental health professionals. Different solutions have used this data set to detect human behavioral patterns and perform association, classification, and prediction of mental states. For example, by using the StudentLife data set, Saeb et al [80] analyzed the correlation between mobility patterns identified from GPS samples and depressive symptoms reported by students. Farhan et al [84] designed a multiview biclustering model using various features (accelerometer, screen state, light, conversation data, and GPS) to identify clusters representing behavior subgroups. Morshed et al [81] developed a computational method to predict mood stability from behavioral features (eg, frequency of conversation, number of location changes, and duration of different physical activities) extracted from accelerometer, microphone, GPS, and Wi-Fi. Recently, de Moura et al [82,83] developed a solution capable of detecting sociability patterns and routine changes in social event streams (ie, conversation events).

A related issue is the predominance of solutions developed for Android OS, for which all apps have a version. This is expected as Android provides an open development platform, different from iOS, with significantly more flexibility to gather the data of interest. The divergent approaches to sensing on iOS and Android yield further issues in terms of standardization and the collection of comparable results across large cohorts, invariably with both Android and iOS users.

Our review further shows that studies use a mix of smartphone-based sensing and wearable device sensing. The latter may be useful where smartphones do not provide quality data (eg, for heart rate, physical activity during sport, or sleep quality), but do pose an issue in terms of interpretability of data given the variety of wearable devices available on the market, each of which use different algorithms. The interpretability of resulting information is further confounded as some of the most popular devices use proprietary algorithms to measure the behaviors of interest or provide aggregate data. Standards would need to consider the commercial pressure for device manufacturers that results in algorithms being proprietary and thus making it difficult to compare information from different devices.

Regarding the year of publication of the studies, most articles (n= 9) have been published in the last 3 years (Figure 7). These data reveal a growing trend in the number of solutions proposed for DPMH.

### Research Opportunities

From this review, we are able to identify different research opportunities for DPMH sensing apps, which are open issues for further investigation.

#### Wearable-Based Solutions

Raw data have been generated mainly in smartphones, so few sensing apps have taken advantage of the potential of wearable devices to produce monitored individual’s data ubiquitously. Wearables are capable of providing a lot of useful information about human behavior [79]. For example, wearable devices such as smartwatches and wristbands can collect users’ context data even when they are performing intense physical activities such as running and swimming. Therefore, as these devices are smaller, meaning more imperceptible to the user, they can enrich the physiological data collection [85].

#### Explainable Models With a Focus on Human Behavior

DPMH sensing apps that perform data analysis to design intelligent models have used traditional ML algorithms in different tasks [20]. These models sometimes lack transparency, which is not helpful for mental health professionals because evidence in decision support tools is required to be explainable. Although traditional ML models are very useful for generating valuable information that supports mental health treatment, an explanation of how they generate their outputs is desirable. This is fundamental because professionals need to interpret the patient’s behavior to perform assessments and interventions. Therefore, explainable models [86] seems to be the way to apply machine and deep learning techniques more suitable to DPMH.

#### Real-Time Inference Engines

Most sensing apps perform offline data analysis after collecting raw data (eg, to create ML models, to correlate self-reports with context data). Therefore, few solutions provide inference engines to produce high-level information in real time. These generated situations of interest are useful to have a better insight into the patient’s behavior and to allow interventions to adapt to this information in real time. This is crucial in extreme cases such as signs of suicidal ideation, but generally useful where the goal is to implement ecological momentary interventions or just-in-time interventions that rely on just-in-time information on user status. In this sense, both rule-based engines (eg, fuzzy logic [39], complex event processing [82]) and ML-based approaches [20] are promising tools to process context data efficiently and infer high-level information in DPMH.

#### Extensible Solutions

Sensing apps are not able to be customized for use in other research. Although general-purpose (eg, Sensus [35]) and reusable (eg, Beiwe [15] and SituMan [39]) apps can be applied to other research, none of the solutions identified in this review is extensible. Proposals of framework, middleware, and library are examples of extensible solutions that provide services, reusable code, and are prepared to be modified or consumed by apps. They would be very useful to allow DPMH researchers to extend solution’s capabilities to different requirements. Therefore, this could reduce costs and time for research in specific scenarios.

By analyzing the results of the public data set review, we clearly identify the scarcity of data sets (n=8). This low number may be related to the privacy of information collected from study participants. DPMH researchers should possibly be concerned about whether collected data will become public, which could enable to identify participants from them. DPMH data sets may have sensitive personal information about the mental health treatment or monitoring, hence ethical issues arise [87]. Moreover, ethics committees where studies are recorded may restrict the sharing of collected data to the public. This barrier can generate great difficulty for the development of new research, because new ML models and engines for inferring high-level information are not possible to be designed and trained. Differential privacy seems to be a promising tool to break this barrier [88].

Another open issue is the standardization of data sets. Currently, there is no standard for data representation (eg, data type, precision, file format) and collection (eg, frequency, duration, presence of time stamps). As a result, data sets cannot be combined, nor can we easily compare the performance of different approaches or algorithms. Proposals for standardization would be a major contribution to the DPMH field.

It is beneficial for such standardization that there are efforts to design general-purpose sensing apps. We propose that the research community should endeavor to work on such apps collaboratively and make these apps available on a non-for-profit basis. This could not only result in an efficient use of commonly agreed standards, but would also reduce the wasteful effort of developing custom sensing apps. Such initiatives, however, are difficult to start and maintain, as has been shown by brave endeavors such as Beiwe [15], Funf [31], Purple Robot [33], and Sensus [35], which show that keeping such platforms up-to-date is an expensive process that can only be warranted if continued use guarantees continued resources for maintenance and further development.

Notwithstanding the benefits we believe would be derived from such standards, it should be acknowledged that self-reports will likely remain an important modality to improve the quality of automatically measured behaviors, or to measure behaviors or states that cannot be automatically measured. An opportunity that is not widely leveraged is using the automatically measured behaviors to trigger such self-reports. This would allow self-reports to be more appropriate to the user’s context, further inform automated measures in case sensor measurements do not provide a clear enough picture, and be less intrusive. Software for such a functionality has been proposed previously [39,89], and we believe such a functionality should be part of standardized tools for capturing DPMH.

Finally, data sets are composed of few study participants. It may be difficult for researchers in attracting participants to the research and, at the same time, making them remain until the end of the study. The low number of participants can potentially compromise the use and validation of some data contained in the data sets, and this directly reflects the use of data sets in other DPMH surveys, where it requires a high number of participants to be validated.

### Limitations and Future Work

A first limitation is that data sets and articles published in languages other than English were not included in this review. Second, the search for sensing apps was restricted to 8 digital libraries, although we searched 20 sources with numerous public data sets. Finally, our review is limited by studies reported in the published literature and data sets available to be downloaded.

In addition, we did not focus on security and privacy aspects of DPMH apps in this review. Therefore, our plans include a systematic analysis on the security and privacy features provided by DPMH apps. As this is an extremely sensitive aspect in the development of new functionalities for current and new DPMH mobile systems, a particular characterization with deeper analysis is required. Therefore, we plan to dedicate efforts on this topic for further investigation.

### Conclusions

In this article, we described a systematic review that resulted in a deep analysis of 31 sensing apps and 8 public data sets for DPMH. Results showed a growth in DPMH sensing apps in recent years as opposed to a scarcity of public data sets. We answered the research questions, then showing, for example, the most used context data and their respective sources, the different types of high-level information generated by the analysis of the collected data, the features available in data sets, and the mental disorders that researchers have focused. From the results, we were able to identify trends and open issues that hinder the development of research in the DPMH area. As a consequence, by considering the growth in proposals for DPMH sensing apps and the impact of the COVID-19 outbreak on global mental health, we believe that DPMH presents a great perspective for future research not only to overcome open issues discussed in this review, but also to reach the needed maturity for application in clinical settings.

## Appendix

Multimedia Appendix 1 List of 20 data set repositories.

Multimedia Appendix 2 Items used in the data extraction process.

Multimedia Appendix 3 Reviewed sensing apps.

## Abbreviations

DPMH: digital phenotyping of mental health
DS-RQs: research questions data sets
EC: exclusion criteria
EMA: ecological momentary assessment
GPS: global positioning system
IC: inclusion criteria
ML: machine learning
PHQ-9: 9-item Patient Health Questionnaire
SA-RQs: research questions for sensing apps
