# Supplementary material for: Sensing Apps and Public Data Sets for Digital Phenotyping of Mental Health: Systematic Review
Source: J Med Internet Res. 2022 Feb 17;24(2):e28735. doi: 10.2196/28735 (PMC8895287; doi:10.2196/28735)
Supplement: Multimedia Appendix 2 [file jmir_v24i2e28735_app2.docx]

**Items used in the data extraction process.**

| **ID** | **Items** |
| --- | --- |
| **Scientific articles** | |
| 1 | Reference (authors, title, and year) |
| 2 | Application name |
| 3 | Operating system (e.g., Android, iOS) |
| 4 | Context data sources (e.g., GPS coordinates, accelerometer, Bluetooth encounters, call logs) |
| 5 | High-level information that can be inferred from collected context data (e.g., sociability, physical activity, and sleep) |
| 6 | State/mental disorder |
| 7 | App purpose |
| 8 | Performed experiments (e.g., number of people in experiments, whether they are patients or healthy people, time and location of the experiment) |
| 9 | Type of analysis (i.e., classification, prediction and recognition of situations of interest, correlation of mental health reports with collected context data, or the gathering of raw context data only) |
| 10 | URL to access the application. |
| **Data sets** | |
| 1 | Title |
| 2 | URL |
| 3 | Description |
| 4 | Participants number |
| 5 | Context data |
| 6 | Device type |
| 7 | Studies published providing or describing the dataset |
| 8 | Category (e.g., sociability, physical activity, sleep, and multimodal) |
