# Supplementary material for: Sensing Apps and Public Data Sets for Digital Phenotyping of Mental Health: Systematic Review
Source: J Med Internet Res. 2022 Feb 17;24(2):e28735. doi: 10.2196/28735 (PMC8895287; doi:10.2196/28735)
Supplement: Multimedia Appendix 3 [file jmir_v24i2e28735_app3.docx]

**Reviewed sensing apps.**

| **Reference** | **OS** | **Context data source** | **Mental disorder** | **High-level information** | **Type of Analysis** |
| --- | --- | --- | --- | --- | --- |
| Funf  [48] (2011) | Android | GPS, Wi-Fi, accelerometer, Bluetooth encounters, cell tower ID, calls, SMS, web browser history, contacts, app installation logs, screen on/off, app usage logs, and battery level | Mental state in general | It does not infer information | Raw data collection |
| Mobilyze [46] (2011) | Android | Accelerometer, altitude, ambient light, battery level, Bluetooth encounters, calendar events, proximity, calls, GPS, email, SMS, ring volume, and screen on/of | Depression | Mood, emotions, cognitive/motivational states, physical activity, social context | Mental state prediction |
| Purple Robot  [49] (2014) | Android | Accelerometer, altimeter, gyroscope, GPS, Wi-Fi, Bluetooth encounters, battery level, calls, SMS, app usage logs, and screen on/of | Mental state in general | It does not infer information | Raw data collection |
| AWARE [50] (2015) | Android / iOS | Accelerometer, barometer, battery level, Bluetooth encounters, calls, SMS, gravity, gyroscope, app installation logs, GPS, Wi-Fi, proximity, screen on/off, and cell tower ID | Mental state in general | It does not infer information | Raw data collection |
| Sensus  [59] (2016) | Android / iOS | Acceleration, altitude, compass, GPS, POI Proximity, social media, call, SMS, ambient temperature, battery level, Bluetooth encounters, cell tower ID, and screen on/of | Mental state in general | It does not infer information | Raw data collection |
| MOSS  [52] (2016) | Android | Time information, GPS, Wi-Fi, screen on/off, Bluetooth encounters, SMS, calls, and calendar events. | Depression | Physical activity, mobility, device usage, sociability, app usage | Mental state classification |
| Beiwe  [15] (2016) | Android / iOS | GPS, accelerometer, calls, SMS, Wi-Fi, Bluetooth encounters, microphone, battery level, and screen on/of | Mental state in general | It does not infer information | Raw data collection |
| EVO  [74] (2016) | Android / iOS | GPS, SMS, calls, screen on/off, and accelerometer | Depression | It does not infer information | Raw data collection |
| CrossCheck [43] (2016) | Android | Microphone, accelerometer, ambient light, GPS, app usage logs, calls, and SMS | Schizophrenia | Sleep, Sociability, mobility, physical activity, device usage | Mental state prediction |
| SituMan [66] (2017) | Android | Accelerometers, time information, and GPS | Mental state in general | Daily routine situations (e.g., working, studying) | It recognizes daily routine situations using fuzzy logic |
| EmotionSense  [56] (2017) | Android | Accelerometer, microphone, GPS, Wi-Fi, SMS, and calls | Mood | Semantic locations, physical activity, sociability | Correlation analysis and mental state classification |
| StudentLife [25] (2017) | Android | Microphone, Bluetooth encounters, GPS, battery level, Wi-Fi, accelerometer, ambient light, and calls | Stress, depression, and loneliness | Sociability, mobility, physical activity, device usage | Correlation analysis |
| *Undefined* [75] (2017) | Android | Microphone, accelerometers, Bluetooth encounters | Mental state in general | Physical activity, mobility and sociability | Correlation analysis |
| AMoSS  [76] (2018) | Android | GPS | Depression | Mobility | Mental state prediction |
| eB2  [77] (2018) | Android | Calls, SMS, app usage logs, GPS, and actigraphy | Depression | Mobility | Mental state classification |
| EARS  [78] (2018) | Android / iOS | GPS, app usage logs, accelerometer, ambient light, screen on/off, SMS, calls, battery level and microphone | Mental state in general | It does not infer information | Raw data collection |
| SleepGuard  [79] (2018) | Android | Accelerometer, gyroscope, microphone, and ambient light | Sleep Disorder | Posture/position body when sleeping | Mental state classification |
| Moment [80] (2018) | iOS | App usage logs | Depression | It does not infer information | Mental state classification |
| TypeOfMood  [81] (2019) | Android | Touch screen | Stress | It does not infer information | Mental state classification |
| RADAR-base  [82] (2019) | Android / iOS | Accelerometer, Bluetooth encounters, battery level, calls, gyroscope, ambient light, GPS, Wi-Fi, field magnetic, SMS, step count (Fitbit), and app usage logs | Mental state in general | It does not infer information | Raw data collection |
| SHADO  [53] (2019) | Android | Accelerometer, GPS, ambient light, and calls | Depression | Physical activity, mobility, sleep, sociability | Correlation analysis and mental state classification |
| InSTIL  [83] (2019) | Android / iOS | Accelerometer, gyroscope, compass, barometer, ambient light, GPS, microphone, camera, Wi-Fi, Bluetooth encounters, app usage logs, calls, SMS, and touch screen | Mental state in general | It does not infer information | Raw data collection |
| Lamp  [84] (2019) | Android | GPS and step counter (Fitbit) | Anxiety, depression, and psychotic symptoms | Physical activity | Correlation analysis |
| SOLVD  [47] (2020) | Android | Accelerometer, GPS, step counter (smartphones), calls, SMS, screen on/off, and ambient light | Depression | Mobility, sociability, context of daily life (e.g., duration of sleep) | Correlation analysis |
| STDD  [55] (2020) | Android | Calls, app usage logs, ambient light, and accelerometer | Depression | Physical activity, mood, sociability, sleep | Mental state classification |
| Moodable [54] (2020) | Android | Microphone, GPS, calls, SMS, web browser history, and app usage logs | Depression and suicidal ideation | Sociability and mobility | Mental state classification |
| Cogito Companion [85] (2020) | Android | GPS, calls, and SMS | Mental state in general | Mood, stress level, and well-being | Mental state classification |
| Strength Within Me [86] (2020) | iOS | Step counter (Fitbit) and app usage logs | Suicidal Ideation | Sleep, mobility, and sociability | Mental state prediction |
| EuStress [44] (2020) | Android | Camera and body temperature | Stress | It does not infer information | Mental state prediction |
| Mood Triggers [87] (2020) | Android | GPS, accelerometer, Wi-Fi, ambient temperature, ambient light, heart rate, and calls | Depression | Mobility and sociability | Mental state prediction |
| Data Collector [88] (2020) | Android | GPS and accelerometer | Depression | Physical activity and mobility | Mental state classification |
